# Sustained Accumulation of Blood-Derived Macrophages in the Immune Microenvironment of Patients with Recurrent Glioblastoma after Therapy

**DOI:** 10.3390/cancers13246178

**Published:** 2021-12-07

**Authors:** Sara Magri, Beatrice Musca, Camilla Bonaudo, Ada Tushe, Maria Giovanna Russo, Elena Masetto, Vittorina Zagonel, Giuseppe Lombardi, Alessandro Della Puppa, Susanna Mandruzzato

**Affiliations:** 1 Department of Surgery, Oncology and Gastroenterology, University of Padova, 35128 Padova, Italy; sara.magri.1@unipd.it (S.M.); ada.tushe@phd.unipd.it (A.T.); 2 Immunology and Molecular Oncology, Veneto Institute of Oncology IOV—IRCCS, 35128 Padova, Italy; beatrice.musca@iov.veneto.it (B.M.); mariagiovanna.russo@iov.veneto.it (M.G.R.); elena.masetto.2@gmail.com (E.M.); 3 Neurosurgery, Department of NEUROFARBA, University Hospital of Careggi, University of Florence, 50134 Florence, Italy; camilla.bonaudo@gmail.com (C.B.); alessandro.dellapuppa@unifi.it (A.D.P.); 4 Medical Oncology Unit 1, Veneto Institute of Oncology IOV—IRCCS, 35128 Padova, Italy; vittorina.zagonel@iov.veneto.it (V.Z.); giuseppe.lombardi@iov.veneto.it (G.L.)

**Keywords:** glioblastoma, macrophages, tumor microenvironment, recurrence/relapse

## Abstract

**Simple Summary:**

Glioblastoma (GBM) is the most aggressive type of brain cancer and, despite standard treatments, recurrence is inevitable. The immunosuppressive microenvironment, characterized by an intense recruitment of myeloid cells and a low frequency of anti-tumor lymphocytes, hampers the success of new immunological therapies. Thus, understanding how treatments impact the tumor microenvironment is crucial to limit recurrences. In this study, we compared the immune profile in the central and in the marginal areas of resected tumors on a cohort of patients with primary or relapsing GBM and identified a different immune composition according to tumor localization. In addition, levels of four subsets of myeloid-derived suppressor cells were determined before and after standard therapy. Significant correlations were obtained by combining data collected from tumor and blood, thus reinforcing the notion that immunosuppression should be evaluated both in the circulation and in the tumor microenvironment, to circumvent this phenomenon from a therapeutic point of view.

**Abstract:**

The cell composition of the glioblastoma (GBM) microenvironment depends on the recruitment of myeloid cells from the blood, promoting tumor progression by inducing immunosuppression. This phenomenon hampers immunotherapies and investigating its complexity may help to tailor new treatments. Peripheral blood and tissue specimens from the central and marginal tumor areas were collected from 44 primary and 19 recurrent GBM patients. Myeloid and lymphoid cell subsets and the levels of immunosuppressive markers were defined by multiparametric flow cytometry. Multiplexed immunohistochemistry was used to confirm the differences in the immune infiltrate and to analyze the cell spatial distribution. Relapsing GBM showed an increased presence of blood-derived macrophages in both tumor areas and a higher frequency of infiltrating lymphocytes, with a high level of exhaustion markers. The expansion of some myeloid-derived suppressor cell (MDSC) subsets in the blood was found in both primary and recurrent GBM patients. A significant inverse correlation between infiltrating T cells and an MDSC subset was also found. In patients with recurrent GBM after standard first-line therapy, the immune-hostile tumor microenvironment and the levels of some MDSC subsets in the blood persisted. Analysis of the immune landscape in GBM relapses aids in the definition of more appropriate stratification and treatment.

## 1. Introduction

New evidence in brain immunity has led to the re-evaluation of the concept of the brain as an *immuno-privileged* organ [1,2,3,4]. In the case of primary brain malignancies, such as glioblastoma (GBM), the disruption of the blood–brain barrier also contributes to the infiltration of a variety of immune cells, attracted by soluble factors produced by the tumor [5]. In particular, it is now clear that GBM tumors share a well-defined pattern of leukocyte infiltration in which bone marrow-derived and immune suppressive macrophages (BMDMs) represent the main cell subset and coexist along with microglia (MG), the resident macrophages of the central nervous system [6]; T cells are scarcely present and show markers of exhaustion, thus leading to the consideration of GBM as a cold tumor [7]. The profound immunosuppression of the tumor microenvironment (TME) represents one of the major challenges in GBM treatment [7], and in recent years the immune microenvironment of gliomas and of recurrences has been studied using cutting-edge technologies [8,9,10,11], with the aim of characterizing and exploiting the TME for new therapies.

Treatment after surgery may impact the immune microenvironment, potentially with adverse or beneficial outcomes, according to the therapy. Studies on a limited number of human GBMs by scRNAseq [10] or mass CyTOF [12] indicate the presence of tumor macrophages as the main immune population in both primary and recurrent GBM, although with different indications regarding the composition and proportion of tumor macrophages in primary and secondary surgery. However, less agreement concerns the infiltrating lymphoid population, with some works showing no difference in lymphocyte proportions between primary and recurrent GBM [12] and others suggesting a greater contribution of lymphocytes [10] or increased frequencies of CD8^+^ T cells in recurrent tumors [13].

Recently, we analyzed the immune microenvironment of gliomas at diagnosis in untreated patients [14]. This analysis disclosed the influx of a large proportion of BMDMs endowed with high immune suppressive activity and located in the central part of the lesion. Instead, in the marginal area we found a reduced presence of BMDMs, with a lower immune suppressive activity on a per cell basis. These findings were obtained through fluorescence-based surgery with 5-aminolevulinic acid (5-ALA) and indicate that the location in the tumor area has an impact on the function exerted by macrophages in the TME. The study of the immune microenvironment at the surgical margin is therefore of relevance, since the infiltrative perilesional lesion represents the starting point of tumor recurrence and potentially the target of subsequent therapies.

Understanding the changes of the immune TME in GBM relapses following treatment may assist in evaluating the impact of therapies on the TME in order to develop novel immunotherapeutic strategies. Thus, the aim of this study is to analyze GBM immune profile of the core and of the margins at diagnosis and/or recurrence at cellular level by multiparametric flow cytometry. In addition, the main immune suppressive myeloid subsets of the peripheral blood of patients were analyzed. Our results indicate a continuous recruitment of blood-derived macrophages in recurrent GBM, together with an increased T cell infiltration, characterized by a high expression of exhaustion markers LAG-3 and PD-1.

## 2. Materials and Methods

### 2.1. Patient Characteristics and Treatment

Patients were recruited at the Department of Neurosurgery of Padova and Florence University Hospital, Italy. Forty-four newly diagnosed and nineteen recurrent GBM patients, whose features are reported in Table 1, underwent tumor resection with the aid of 5-ALA-assisted surgery. Overall, 99 tumor specimens were obtained from the central fluorescent area (37 from primary and 17 from recurrent tumors) and from the marginal dimly fluorescent zone (37 from primary and 8 from relapsed patients) and 58 blood samples (43 before first surgery and 15 before second surgery) were collected and processed. Matched samples with first surgery and relapse were obtained from 4 patients. Peripheral blood was also drawn from 14 healthy donors (HD) (Table 1).

All the experiments were approved by the ethical committee of Veneto Institute of Oncology IRCCS of Padova, Italy (MDSC_SNC 2016/13), and of Padova and Florence University Hospital (NOI_NCH 1536/19) and all patients gave their written informed consent. The study was carried out in line with the Declaration of Helsinki.

All the patients enrolled in this study were treated after surgery at Veneto Institute of Oncology; all patients received standard first-line treatment of concomitant radiochemotherapy with temozolomide followed by maintenance temozolomide. Neuroradiological assessment was based on RANO criteria.

### 2.2. Blood and Tumor Sample Processing

Peripheral blood was collected from tumor patients immediately prior to surgery and labelled for multiparametric flow cytometry within two hours after withdrawal. All tumor specimens were immediately processed after resection, with the exception of those provided by a neurosurgery in Florence, which were kept in MACS^®^ Tissue Storage Solution (Miltenyi Biotec, Bergisch Gladbach, Germany) at 4 °C and processed the day after collection. GBM specimens were firstly washed with 0.9% sodium chloride to remove peripheral blood traces and then digested mechanically and enzymatically by using the Tumor Dissociation Kit (Miltenyi Biotec) and the gentleMACS™ Octo Dissociator (Miltenyi Biotec) according to the manufacturer’s instructions for soft tumors.

### 2.3. Multiparametric Flow Cytometry

Fresh unrefrigerated whole blood was stained with monoclonal antibodies to analyze myeloid cell subsets, as previously described in [15]. MDSC cell subsets were identified by seven-color staining with the following antibodies: anti-CD11b Alexa700 (BD Biosciences, Becton Dickinson, Franklin Lakes, NJ, USA), anti-CD14 APC-H7 (BD Biosciences), anti-CD15 V450 (BD Biosciences), anti-CD33 PE-Cy7 (eBioscience, Thermo Fisher Scientific, Waltham, MA, USA), anti-IL4Rα PE (R&D SYSTEMS, Minneapolis, MN, USA), lineage cocktail (Lin) FITC (BD Biosciences) and anti-HLA-DR APC (BD Biosciences). The immunophenotyping was standardized as described below. Single-cell suspensions from GBM specimens were stained with different antibody mixtures for characterizing myeloid and lymphoid cell subsets as previously described [14]. Antibody mixtures contained: LIVE/DEAD™ Fixable Aqua (Life Technologies, Thermo Fisher Scientific), anti-CD45 BV421 (BD Biosciences), anti-CD33 PE-Cy7 (eBioscience) or anti-CD33 APC (BD Biosciences), anti-HLA-DR APC (BD Biosciences), anti-CD3 PE-Cy7 (Beckman Coulter, Indianapolis, Indiana, USA), anti-CD8 APC-H7 (BD Biosciences), anti-LAG-3 FITC (AdipoGen, San Diego, CA, USA), anti-PD-1 PE (Miltenyi Biotec) and anti-CD49d PE (BioLegend, San Diego, CA, USA). Fluorescence minus one (FMO) tubes for PD-1 and LAG-3 were prepared as negative controls.

Data acquisition was carried out with BD™ LSRII flow cytometer (BD Biosciences). For data analysis, FlowJo software (Becton Dickinson, Franklin Lakes, NJ, USA) was used.

### 2.4. Standardization of MDSC Staining Acquisition and Analysis

The standardization of MDSC staining was carried out using a dilution of antibodies, maximizing the signal to noise ratio upon titration of the single antibodies. In parallel to the staining of the blood from each patient, the performance of anti-HLA-DR and anti-IL4Rα antibodies was monitored by staining a batch of fixed and permeabilized EBV-B cell line, constitutively expressing these markers at high intensity, and by performing their acquisition before proceeding with patient blood samples. This allowed us to evaluate whether the mean fluorescence intensity (MFI) of the two markers fell in the range of tolerance previously built through repeated staining of the cell line. Moreover, FMO tubes for HLA-DR and IL4Rα were prepared as negative controls for each blood sample. The performance of the BD™ LSRII flow cytometer (BD Biosciences) was also assessed by following the protocol described by Perfetto et al. [16].

### 2.5. Multispectral Imaging

Formalin-Fixed Paraffin-Embedded (FFPE) specimens from one patient both at diagnosis and recurrence were analyzed by multispectral imaging. FFPE slides were stained with anti-CD8 (clone C8/144B, Thermo Fisher Scientific, Waltham, MA, USA), anti-CD68 (clone PG-M1, Dako Agilent, Santa Clara, CA, USA) and anti-glial fibrillary acidic protein (GFAP, clone EP672Y, abcam, Cambridge, UK) antibodies for T cell, macrophage and surrounding tissue detection, respectively. DAPI (Akoya Biosciences, Marlborough, MA, USA) was used as a nuclear counterstain. The autofluorescence background signal was subtracted using an unstained control slide processed in parallel. The Mantra multispectral imaging platform (Akoya Biosciences) was employed for image acquisition at 20× magnification and data analysis was carried out with InForm 2.4.1 software (Akoya Biosciences). The immune cell infiltrate was quantified as cell density/megapixel.

### 2.6. Cell Sorting and Cytospin Preparation

Live CD45^+^/CD33^+^/HLA-DR^+^ (BMDMs), live CD45^+^/CD33^+^/HLA-DR^low^ (MG), live CD45^+^/CD33^dim^/HLA-DR^−^ (PMNs), live CD45^+^/CD3^+^ (lymphocytes) and live CD45^−^ (tumor cells) cell subsets were separated by fluorescence-activated cell sorting (FACS, MoFlo Astrios, Beckman Coulter). The purity of each fraction was >90%. After cell sorting, cytospins were prepared by centrifuging cells on microscope slides and carrying out May-Grünwald-Giemsa (MGG) staining and analysis as previously described in [17].

### 2.7. Statistical Analysis

Data statistical analysis was performed with SigmaPlot software (Systat Software Inc., San Jose, CA, USA) by using the Mann–Whitney test and Student t-test; all tests were two sided and variations were considered statistically significant with *p*-value < 0.05. The lack of significance was not reported for brevity. Spearman’s correlation was performed to assess the correlation between different parameters.

## 3. Results

### 3.1. Immune Infiltration in Primary versus Relapsing GBM in Different Tumor Areas

**Figure 1:**
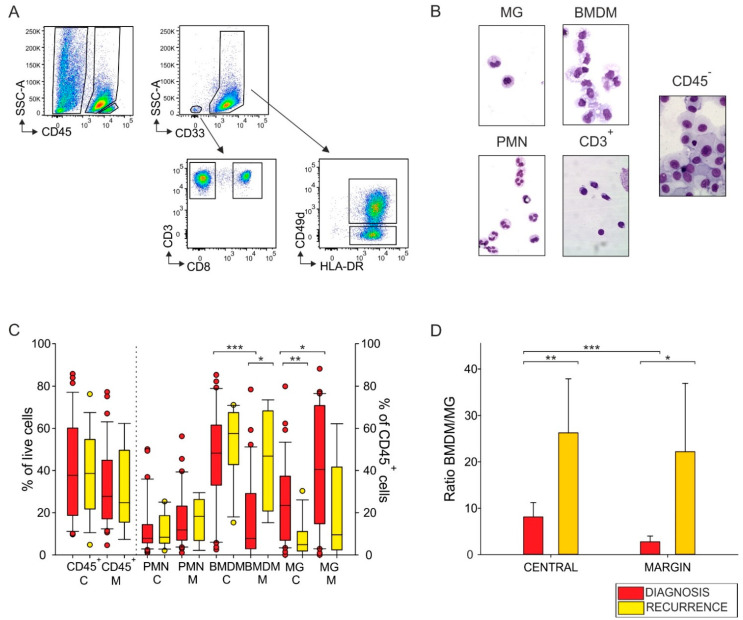
Distribution of tumor-infiltrating leukocytes in primary and recurrent glioblastoma (GBM) patients. (**A**) Representative flow cytometry analysis for myeloid and lymphoid cell subset identification in tumor specimens. CD45^+^ cells were identified among live cells, after morphological evaluation and doublets exclusion; polymorphonuclear (PMN) cells, macrophage subsets CD33^high^/HLA-DR^+^/CD49d^+^ (bone marrow-derived macrophages, BMDMs) and CD33^high^/HLA-DR^+^/CD49d^−^ (microglia, MG) and CD3^+^ lymphocytes were gated among CD45^+^ leukocytes, while CD8^+^ and CD4^+^ subsets were gated on CD3^+^ cells. (**B**) Different cell populations were purified by fluorescence-activated cell sorting (FACS) and May-Grünwald-Giemsa (MGG) stained. PMNs were identified as CD33^dim^/HLA-DR^−^ and tumor cells as CD45^−^. (**C**) Box plots show the median, 25th and 75th percentile of the frequency of tumor-infiltrating leukocytes in the central (C) and in the marginal (M) areas of primary (red) and recurrent (yellow) GBM tumors; whiskers extend to 1.5 inter-quartile range and outliers are shown by dots (*n* = 37 for CD45^+^ and *n* = 31 for BMDMs and MG from central layer of GBM; *n* = 17 for CD45^+^ and *n* = 12 for BMDMs and MG from central layer of recurrences; *n* = 37 for CD45^+^ and *n* = 29 for BMDMs and MG from marginal layer of GBM; *n* = 7 for CD45^+^ and *n* = 5 for BMDMs and MG from marginal layer of recurrences). (**D**) Bars represent the median ratios between the percentages of BMDMs and MG in 33 central and 30 marginal GBM specimens and in 9 central and 6 marginal relapsing ones. Two-tailed Mann-Whitney test, * *p* < 0.05, ** *p* < 0.01, *** *p* < 0.001.

**Figure 2:**
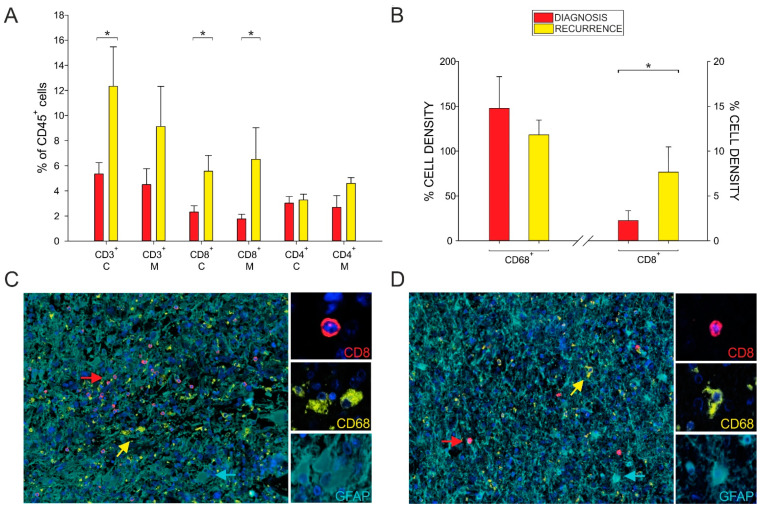
Analysis of the presence of immune infiltrate. (**A**) Analysis of T cell populations by multiparametric flow cytometry. Bars represent the percentages of lymphocytes in the central (C) and marginal (M) layers, in primary (red) and recurrent (yellow) GBM patients (central layer: *n* = 12 for GBM and *n* = 9 for recurrences; marginal area: *n* = 16 for GBM and *n* = 3 for recurrences). (**B**) Analysis of the immune infiltrate by multispectral imaging. Cell infiltrate in tumor specimens was quantified as cell density per megapixel. Bar plots show the mean ± SE of the cell density per megapixel of CD68^+^ and CD8^+^ cells calculated by considering 12 fields for primary (red plots) and 11 fields for recurrent GBM (yellow plots). Two-tailed Mann-Whitney test, * *p* < 0.05. (**C**,**D**) Representative images of multispectral fields (20× zoom) and corresponding cell type identification of macrophages (CD68^+^ cells, in yellow), CD8^+^ cells (in red) and tumor (GFAP^+^ cells, in light blue) from a patient with GBM (**C**) and its recurrence (**D**). DAPI (blue) was used as a nuclear counterstain.

In this study, we enrolled 44 patients with newly diagnosed GBM and 19 with recurrent GBM, whose molecular characteristics are shown in Table 1. To evaluate the main myeloid and lymphoid cell subsets present in the GBM microenvironment, we analyzed both the central and marginal areas at diagnosis and at relapse using intraoperative tumor visualization with fluorescence-guided surgery by 5-ALA imaging. The highly fluorescent and the dimly fluorescent areas corresponded to the central non-necrotic and to the marginal area, respectively. Analysis of the immune infiltrate was performed with multiparametric flow cytometry, and a representative example of the gating strategy used is reported in Figure 1A. After doublets exclusion and gating on live cells, we first determined the leukocyte cell abundance by analyzing the percentage of CD45^+^ cells (Figure 1A) and found a median ranging from 24.8% to 38.6% of live cells across the tumor samples, without significant differences between diagnosis and relapses in either of the tumor areas (Figure 1C). We then focused on myeloid and T cell infiltrate, given their opposing roles in the immune response against the tumor. Myeloid cells were identified as blood-derived or resident macrophages (BMDMs and MG, respectively) by using CD49d cell marker, and also as polymorphonuclear cells (PMNs), while CD3^+^ T cells were further divided into CD8^+^ and CD8^−^ (i.e., CD4^+^) T cells (Figure 1A). Collectively, BMDMs, MG, PMNs, CD4^+^ and CD8^+^ cells represent the main immune cell populations of the TME in gliomas [14,18] and we analyzed their presence, in relative proportion, as a percentage of CD45^+^ cells (Figure 1C,D and Figure 2A). As well as phenotyping, to document their identity, we purified several leukocyte subsets and tumor cells (identified as CD45^−^ cells) by cell sorting and confirmed their identity by morphology (Figure 1B). In line with our previous results, we observed a higher presence of BMDMs in the central area of newly diagnosed GBM than in the marginal part of the tumor, while we noted the opposite distribution for MG cells (Figure 1C) [14]. In relapsing GBM, we instead found that the presence of tumor macrophages redistributed in the two areas, as the presence of BMDMs increased in the marginal area (Figure 1C, *p* = 0.013) and MG decreased in the central zone (Figure 1C, *p* = 0.002). By analyzing the BMDM/MG ratio, we found that it increased in both the central and marginal areas of the tumor (Figure 1D, central area: *p* = 0.008, marginal area: *p* = 0.015). These results suggest that there is an incessant recruitment of suppressive BMDMs in relapsing GBM, despite therapy. However, this phenomenon does not occur for MG cells, highlighting the differences among resident and blood-derived macrophages not only in their immune suppressive ability [14], but also in terms of attraction in the newly formed relapsing tumors.

Notably, we also observed a significant increase of CD8^+^ T cells in both areas of the tumor (Figure 2A). To confirm this result, we analyzed a small number of samples obtained from the central tumor area of a patient both at diagnosis and at relapse through multispectral staining. We found a higher occurrence of CD8^+^ T cells (Figure 2B, *p* = 0.015), confirming the flow cytometry results shown in Figure 2A. In addition, we observed that, although CD8^+^ T cells were more frequent in relapsed patients than recently diagnosed patients, they were sparse in the tumor area, had a rounded shape typical of resting T cells, and lacked interaction with CD68^+^ macrophages (Figure 2C,D).

### 3.2. Evaluation of the Dysfunctional Markers of T Cells

**Figure 3:**
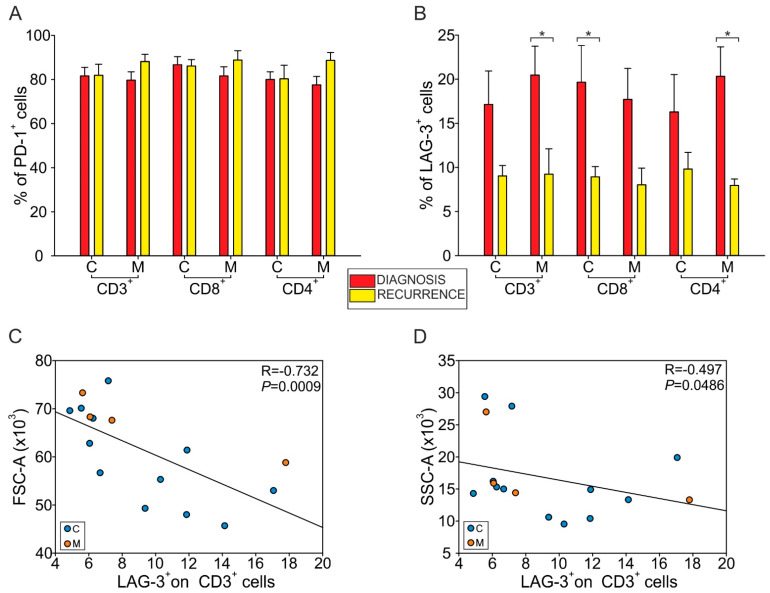
Characterization of the markers of lymphocyte dysregulation on T cells. (**A**) Percentage of CD3^+^, CD3^+^/CD8^+^ and CD3^+^/CD8^−^ (i.e., CD4^+^) cells expressing PD-1 in central (C) and marginal (M) region of primary (red) and recurrent (yellow) GBM (central layer: *n* = 12 for GBM, *n* = 10 for CD3^+^ REC, *n* = 9 for CD3^+^/CD8^+^ and CD3^+^/CD8^−^ cells REC; marginal layer: *n* = 15 for GBM, *n* = 4 for REC). (**B**) Percentage of CD3^+^, CD3^+^/CD8^+^ and CD3^+^/CD8^−^ (CD4^+^) cells expressing LAG-3 in central (C) and marginal (M) region of primary (red) and recurrent (yellow) GBM (central layer: *n* = 12 for GBM, *n* = 11 for CD3^+^ REC, *n* = 9 for CD3^+^/CD8^+^ and CD3^+^/CD8^−^ cells REC; marginal layer: *n* = 15 for GBM, *n* = 4 for REC). Two-tailed Mann-Whitney test, * *p* < 0.05. (**C**,**D**) Correlation between LAG-3 expression on CD3^+^ cells and FSC (**C**) and SSC (**D**). Spearman’s rank-order correlation on 16 paired samples.

To gain insight into the lymphocyte functional response, we analyzed the expression of checkpoint molecules PD-1 and LAG-3 on CD3^+^ T cells in the two areas of GBM specimens, both at diagnosis and at relapse, and observed a steady high frequency of T cells expressing PD-1 in both cases, but a lower expression of LAG-3 on T cells in relapsing tumors (Figure 3A,B). The reduction of LAG-3 expression on CD3^+^ cells was found to be significantly lower in relapses only in the marginal area (Figure 3B, M: 20.46% at diagnosis, 9.22% at relapse, *p* = 0.04; C: 17.13% at diagnosis, 9.03% at relapse, ns); a reduced expression of LAG-3 was on CD8^+^ T cells of the central area of relapsing GBM (Figure 3B, 19.65% in newly diagnosed GBM, 8.93% in recurrences, *p* = 0.036), while the highest reduction of LAG-3 expression was observed on CD4^+^ T cells present in the marginal zone (Figure 3B, 20.32% at diagnosis, 7.95% at relapse, *p* = 0.011).

When we analyzed the correlation between LAG-3 expression on CD3^+^ cells and the forward (FSC-A) and side scatter (SSC-A) of morphological parameters, we observed an inverse correlation only in relapses (Figure 3C, R = −0.732, *p* = 0.0009; Figure 3D, R = −0.497, *p* = 0.0486) but not in primary GBM (Figure S1A,B). As the two morphological parameters refer to cellular size and complexity, these findings suggest that in relapsing tumors a higher expression of LAG-3 is characteristic of small (FSC-A^low^) resting (SSC-A^low^) T cells (Figure 3C,D).

### 3.3. Evaluation of Myeloid Cell Subsets in the Peripheral Blood of GBM Patients after Treatment

**Figure 4:**
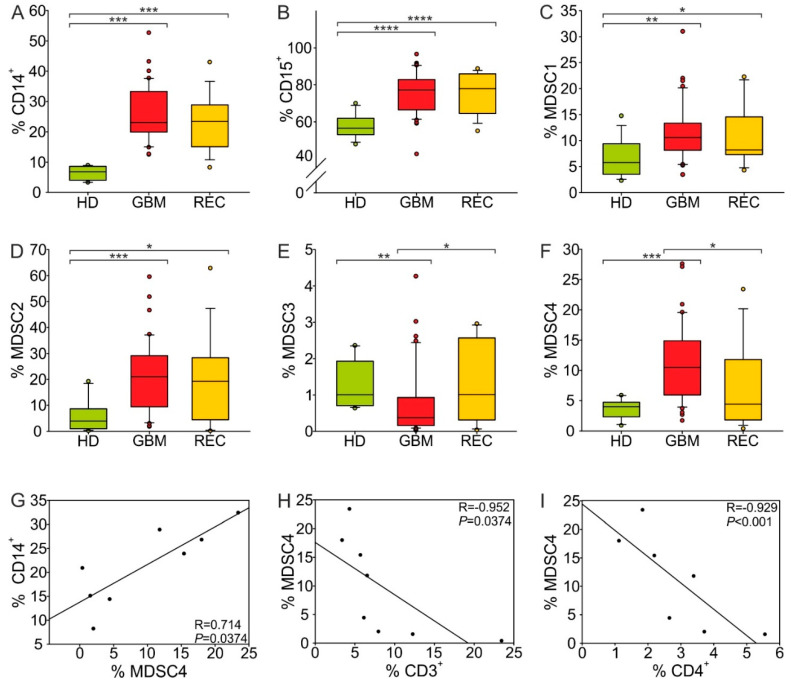
Distribution of circulating myeloid cells in primary GBM and recurrences. Pairwise comparison of cell-associated markers between healthy donors (HDs) and primary/recurrent GBM patients was performed using a two-tailed Mann-Whitney test. (**A**–**F**) Box plots show the median, 25th and 75th percentile of the percentage of (**A**) monocytes, evaluated as CD14^+^ cells among peripheral blood mononuclear cells (PBMCs, *n* = 14 for HDs, *n* = 42 for GBM and *n* = 15 for recurrences), (**B**) PMNs, evaluated as CD15^+^ cells among peripheral blood leukocytes (*n* = 14 for HDs, *n* = 43 for GBM and *n* = 15 for recurrences), (**C**) MDSC1, evaluated as CD14^+^/IL4Rα^+^ cells among PBMCs (*n* = 14 for HDs, *n* = 43 for GBM and *n* = 15 for recurrences), (**D**) MDSC2, evaluated as CD15^+^/IL4Rα^+^ cells among PMNs (*n* = 14 for HDs, *n* = 43 for GBM and *n* = 14 for recurrences), (**E**) MDSC3, evaluated as Lin^−^/HLA-DR^−^/CD11b^+^/CD33^+^ cells in CD15^−^ cells (*n* = 14 for HDs, *n* = 43 for GBM and *n* = 15 for recurrences), (**F**) MDSC4, evaluated as CD14^+^/HLA-DR^low/−^ cells among PBMCs (*n* = 14 for HDs, *n* = 43 for GBM and *n* = 15 for recurrences). Whiskers extend to 1.5 inter-quartile range and outliers are shown by dots. Only statistically significant comparisons are reported: **** *p* < 0.0001, *** *p* < 0.001, ** *p* < 0.01, * *p* < 0.05. (**G**) Correlation between the percentages of monocytes and MDSC4 among PBMCs in recurrent patients. Spearman’s rank-order correlation on 8 paired samples. (**H**,**I**) Correlation between the percentage of circulating MDSC4 and either tumor-infiltrating CD3^+^ cells (**H**) or tumor-infiltrating CD4^+^ cells (**I**). Spearman’s rank-order correlation on 8 (**H**) and 7 (**I**) paired samples, respectively.

Recently, we assessed the prognostic and predictive value of suppressive myeloid subsets in the peripheral blood of glioma patients [19]. Here, we analyze the presence of monocytes (evaluated as CD14^+^ cells), PMNs (CD15^+^ cells) and four myeloid-derived suppressor cell (MDSC) subsets (MDSC1, CD14^+^/IL4Rα^+^; MDSC2, CD15^+^/IL4Rα^+^; MDSC3, Lin^−^/HLA-DR^−^/CD11b^+^/CD33^+^; MDSC4, CD14^+^/HLA-DR^−^) in relapsing GBM patients, after standard treatment. All patients underwent surgical intervention for relapse after standard radiotherapy and chemotherapy treatment, according to the Stupp protocol. As shown in Figure 4A–F, compared to a group of age-matched HDs, monocytes, PMNs, MDSC1, 2 and 4 were significantly increased in primary GBM (monocytes: *p* < 0.001, PMNs: *p* < 0.001, MDSC1: *p* = 0.002, MDSC2: *p* = 0.023, MDSC4: *p* < 0.001), while MDSC3 levels decreased (*p* = 0.002). At relapse, following treatment, the expansion of MDSC1 and 2 remained stable in comparison to levels before a patient’s first surgery, indicating that the recruitment of bone marrow-derived myeloid subsets associated with negative outcome persisted and was unaffected by therapy. Conversely, MDSC3 levels tended to increase (*p* = 0.053) and MDSC4 decreased (*p* = 0.025). In addition, CD14^+^ levels in recurrent GBM were positively correlated with MDSC4 (Figure 4G, R = 0.714, *p* = 0.0374), thus reinforcing the notion that the expansion of monocytes in these patients is associated with an immune suppressive trait. In support of this hypothesis, we also observed that the expansion of MDSC4 in the blood of relapsing GBM correlated negatively with CD3^+^ (Figure 4H, R = −0.952, *p* = 0.0374) and CD4^+^ (Figure 4I, R = −0.929, *p* < 0.001) T cells infiltrating the TME. All the other correlations that were not significant were reported as Figure S2A–M.

## 4. Discussion

GBM is an aggressive disease and nearly all patients relapse. Surgery represents the first modality of treatment and fluorescence-guided surgery with 5-ALA, typically employed for primary high-grade tumor resection, is also useful and effective in recurrent GBM [20,21], even though the pattern of fluorescence may be different to that of primary GBM.

After surgery, the first-line treatment consists of concomitant radiochemotherapy followed by chemotherapy with temozolomide according to the Stupp protocol. Although some new drugs have shown interesting activity [22], treatments following recurrence are limited and lack efficacy [22,23,24,25] due to the main characteristics of GBM relapses, such as intraparenchymal localization of the lesion, molecular heterogeneity and the immunosuppressive mechanisms implemented by the tumor itself. In particular, the presence of a highly immune suppressive microenvironment, mainly established by blood-derived macrophages and by the phenomenon of T-cell exhaustion, is one of the major obstacles to GBM treatment; therefore, exploring the TME is crucial for the identification of new efficient therapies, to improve the outcomes for patients. Given the difficulty of identifying a successful therapy for GBM and the almost inevitable tumor relapse, understanding the immune composition at recurrence may be of help to elucidate the effect of current therapies. This tool should be considered when new treatments become available. The aim of our work was to evaluate the changes characterizing the relapse setting. In line with our previous studies, showing a differential spatial distribution as well as functional activity of myeloid cells in primary GBM [14], we observed that the heterogeneous immune profile of different tumor areas typical of GBM at diagnosis was also maintained in relapses. In addition, we found an overall increased presence of BMDMs in relapsing tumors, especially in the marginal area (Figure 1C). The increased recruitment of blood-derived macrophages to the tumor, despite the therapy, is in line with studies showing a high contribution of monocytes in recurrent GBM human samples [10] as well as with works suggesting that radiation can increase tumor hypoxia, triggering a cascade of events that finally leads to high recruitment of monocytes, thus promoting tumor recurrence [26]. In addition, a study in glioma mouse models showed that ionizing radiation changes not only the abundance, but also the phenotype of MG and BMDMs, inducing radiation-specific gene signatures that can be impaired by targeting them with a colony-stimulating factor 1 receptor (CSF-1R) inhibitor, thus limiting tumor relapse [27].

In terms of the presence of lymphocytes, we found an increased frequency of CD8^+^ T cells, both in the central and marginal areas of tumor relapses (Figure 2A), a finding confirmed by multispectral imaging and reported by others [13]. All the patients in our study underwent radiochemotherapy with temozolomide treatment following surgery, and it is well known that this alkylating drug induces hypermutations in gliomas by alteration of mismatch repair system [28], with subsequent increased mutational load [29]. This, in turn, correlates with higher levels of neoantigens, which is the prerequisite for an adaptive immune response. It is therefore not surprising that in GBM recurrences, there is an increased presence of T cells; however, this infiltration does not translate into an antitumor immune response, given the presence of markers of exhaustion at high levels in relapsing tumors (Figure 3A,B). This suggests a lack of activation, a finding also confirmed by the morphology of resting T cells observed in the tissue (Figure 3C,D). This observation is in line with results from other studies, showing a high PD-1 expression not only on tumor-infiltrating T cells, but also on peripheral blood lymphocytes from recurrent tumors [13], suggesting a systemic exhaustion in relapsing GBM. We concluded at a peripheral level that the increased frequency of monocytes, PMNs and two MDSC subsets observed at diagnosis were maintained at recurrence (Figure 4A–D), while the levels of MDSC4 were decreased in relapsed patients, suggesting their potential use to follow up GBM patients and to monitor relapses, in addition to neuroradiological examination.

In a previous work, we described an unusual case of a patient with a synchronous GBM lesion in the left temporal lobe and a low-grade glioma in the right frontal lobe and followed the evolution of the GBM lesion in two subsequent recurrences. The microenvironment of the GBM lesion had a well-known pattern of BMDM infiltration, concentrated in the central area of the tumor, that steadily maintained during two relapses and following treatments, while the low-grade lesion had essentially only MG cells in the central part of the lesion [30]. Results presented in this work extend these findings to a relatively large cohort of GBM recurrences, showing that the immune suppressive BMDMs dominate the TME of relapses, despite therapy.

## 5. Conclusions

Overall, our findings indicate that the negative role of myeloid cells in GBM also extends to relapsing tumors. Therefore, an effective immunotherapeutic approach in GBM patients should combine the induction of an adaptive immune response with the elimination of myeloid immunosuppressive cells. The identification of tumor-derived factors that contribute to the expansion of suppressive myeloid populations in these patients is thus a topic that, with further research, could lead to more specific and effective treatments.

## Figures and Tables

**Table 1:** Participant characteristics.

Variable	Participant Characteristics				
	Healthy Donors	Newly Diagnosed GBM		GBM Recurrences	
	Blood	Blood	Tumor	Blood	Tumor
Patients, *n*	14	43	44	15	19
Sex					
Male, *n*	10	28	29	9	13
%	71	65	66	60	68
Female, *n*	4	15	15	6	6
%	29	35	34	40	32
Median Age	56	60	59	57	55
Range	36–69	28–79	28–79	41–75	37–75
IDH status					
WT, *n*	NA	39	40	15	17
%	-	91	91	100	89
Mutated, *n*	NA	4	4	0	0
%	-	9	9	0	0
Missing, *n*	NA	0	0	-	2
MGMT status					
Methylated, *n*	NA	24	23	8	8
%	-	56	52	53	42
Unmethylated, *n*	NA	17	20	7	10
%	-	40	45	47	53
Missing, *n*	NA	2	2	0	1

## Data Availability

The data presented in this study are available on request from the corresponding author.

## Supplementary Materials

The following are available online at https://www.mdpi.com/article/10.3390/cancers13246178/s1, Figure S1: Correlation between LAG-3 expression and morphological parameters in T cells infiltrating GBM TME, Figure S2: Correlation between circulating myeloid subsets and tumor-infiltrating lymphocytes in relapsing GBM.

## References

[1] Absinta M., Jonathan K., Nair G., Sati P., Luciano N.J., Palisoc M., Louveau A., Zaghloul K.A., Pittaluga S., Kipnis J. (2017). Human and nonhuman primate meninges harbor lymphatic vessels that can be visualized noninvasively by MRI. eLife.

[2] Brioschi S., Wang W.-L., Peng V., Wang M., Shchukina I., Greenberg Z.J., Bando J.K., Jaeger N., Czepielewski R.S., Swain A. (2021). Heterogeneity of meningeal B cells reveals a lymphopoietic niche at the CNS borders. Science.

[3] Cugurra A., Mamuladze T., Rustenhoven J., Dykstra T., Beroshvili G., Greenberg Z.J., Baker W., Papadopoulos Z., Drieu A., Blackburn S. (2021). Skull and vertebral bone marrow are myeloid cell reservoirs for the meninges and CNS parenchyma. Science.

[4] Louveau A., Smirnov I., Keyes T.J., Eccles J.D., Rouhani S.J., Peske J.D., Derecki N.C., Castle D., Mandell J.W., Lee K.S. (2015). Structural and functional features of central nervous system lymphatic vessels. Nature.

[5] Hambardzumyan D., Gutmann D.H., Kettenmann H. (2016). The role of microglia and macrophages in glioma maintenance and progression. Nat. Neurosci..

[6] Quail D.F., Joyce J.A. (2017). The Microenvironmental Landscape of Brain Tumors. Cancer Cell.

[7] Sampson J.H., Gunn M.D., Fecci P.E., Ashley D.M. (2020). Brain immunology and immunotherapy in brain tumours. Nat. Rev. Cancer.

[8] Darmanis S., Sloan S.A., Croote D., Mignardi M., Chernikova S., Samghababi P., Zhang Y., Neff N., Kowarsky M., Caneda C. (2017). Single-Cell RNA-Seq Analysis of Infiltrating Neoplastic Cells at the Migrating Front of Human Glioblastoma. Cell Rep..

[9] Friebel E., Kapolou K., Unger S., Núñez N.G., Utz S., Rushing E.J., Regli L., Weller M., Greter M., Tugues S. (2020). Single-Cell Mapping of Human Brain Cancer Reveals Tumor-Specific Instruction of Tissue-Invading Leukocytes. Cell.

[10] Pombo Antunes A.R., Scheyltjens I., Lodi F., Messiaen J., Antoranz A., Duerinck J., Kancheva D., Martens L., de Vlaminck K., van Hove H. (2021). Single-cell profiling of myeloid cells in glioblastoma across species and disease stage reveals macrophage competition and specialization. Nat. Neurosci..

[11] Wang L.-B., Karpova A., Gritsenko M.A., Kyle J.E., Cao S., Li Y., Rykunov D., Colaprico A., Rothstein J.H., Hong R. (2021). Proteogenomic and metabolomic characterization of human glioblastoma. Cancer Cell.

[12] Fu W., Wang W., Li H., Jiao Y., Huo R., Yan Z., Wang J., Wang S., Wang J., Chen D. (2020). Single-Cell Atlas Reveals Complexity of the Immunosuppressive Microenvironment of Initial and Recurrent Glioblastoma. Front. Immunol..

[13] Mohme M., Schliffke S., Maire C.L., Rünger A., Glau L., Mende K.C., Matschke J., Gehbauer C., Akyüz N., Zapf S. (2018). Immunophenotyping of Newly Diagnosed and Recurrent Glioblastoma Defines Distinct Immune Exhaustion Profiles in Peripheral and Tumor-infiltrating Lymphocytes. Clin. Cancer Res..

[14] Pinton L., Masetto E., Vettore M., Solito S., Magri S., D’Andolfi M., Del Bianco P., Lollo G., Benoit J.-P., Okada H. (2019). The immune suppressive microenvironment of human gliomas depends on the accumulation of bone marrow-derived macrophages in the center of the lesion. J. Immunother. Cancer.

[15] Damuzzo V., Solito S., Pinton L., Carrozzo E., Valpione S., Pigozzo J., Giancristofaro R.A., Chiarion-Sileni V., Mandruzzato S. (2016). Clinical implication of tumor-associated and immunological parameters in melanoma patients treated with ipilimumab. OncoImmunology.

[16] Perfetto S.P., Ambrozak D., Nguyen R., Chattopadhyay P., Roederer M. (2006). Quality assurance for polychromatic flow cytometry. Nat. Protoc..

[17] Solito S., Falisi E., Diaz-Montero C.M., Doni A., Pinton L., Rosato A., Francescato S., Basso G., Zanovello P., Onicescu G. (2011). A human promyelocytic-like population is responsible for the immune suppression mediated by myeloid-derived suppressor cells. Blood.

[18] Klemm F., Maas R.R., Bowman R.L., Kornete M., Soukup K., Nassiri S., Brouland J.-P., Iacobuzio-Donahue C.A., Brennan C., Tabar V. (2020). Interrogation of the Microenvironmental Landscape in Brain Tumors Reveals Disease-Specific Alterations of Immune Cells. Cell.

[19] Del Bianco P., Pinton L., Magri S., Canè S., Masetto E., Basso D., Padovan M., Volpin F., D’Avella D., Lombradi G. (2021). A liquid biopsy-based approach identifies myeloid cells, STAT3 and arginase-1 as predictors of glioma risk score and patients’ survival. Res. Sq..

[20] Broekx S., Weyns F., De Vleeschouwer S. (2020). 5-Aminolevulinic acid for recurrent malignant gliomas: A systematic review. Clin. Neurol. Neurosurg..

[21] Chohan M.O., Berger M.S. (2019). 5-Aminolevulinic acid fluorescence guided surgery for recurrent high-grade gliomas. J. Neuro Oncol..

[22] Lombardi G., De Salvo G.L., Brandes A.A., Eoli M., Rudà R., Faedi M., Lolli I., Pace A., Daniele B., Pasqualetti F. (2019). Regorafenib compared with lomustine in patients with relapsed glioblastoma (REGOMA): A multicentre, open-label, randomised, controlled, phase 2 trial. Lancet Oncol..

[23] Birzu C., French P., Caccese M., Cerretti G., Idbaih A., Zagonel V., Lombardi G. (2020). Recurrent Glioblastoma: From Molecular Landscape to New Treatment Perspectives. Cancers.

[24] Lombardi G., Idbaih A., Le Rhun E., Preusser M., Zagonel V., French P. (2020). A New Landscape for Systemic Pharmacotherapy of Recurrent Glioblastoma?. Cancers.

[25] Weller M., Cloughesy T., Perry J.R., Wick W. (2013). Standards of care for treatment of recurrent glioblastoma—Are we there yet?. Neuro-Oncology.

[26] Brown J.M. (2020). Radiation Damage to Tumor Vasculature Initiates a Program That Promotes Tumor Recurrences. Int. J. Radiat. Oncol..

[27] Akkari L., Bowman R.L., Tessier J., Klemm F., Handgraaf S.M., de Groot M., Quail D.F., Tillard L., Gadiot J., Huse J.T. (2020). Dynamic changes in glioma macrophage populations after radiotherapy reveal CSF-1R inhibition as a strategy to overcome resistance. Sci. Transl. Med..

[28] Caccese M., Ius T., Simonelli M., Fassan M., Cesselli D., DiPasquale A., Cavallin F., Padovan M., Salvalaggio A., Gardiman M.P. (2020). Mismatch-Repair Protein Expression in High-Grade Gliomas: A Large Retrospective Multicenter Study. Int. J. Mol. Sci..

[29] Daniel P., Sabri S., Chaddad A., Meehan B., Jean-Claude B., Rak J., Abdulkarim B.S. (2019). Temozolomide Induced Hypermutation in Glioma: Evolutionary Mechanisms and Therapeutic Opportunities. Front. Oncol..

[30] Mandruzzato S., Pinton L., Masetto E., Vettore M., Bonaudo C., Lombardi G., Della Puppa A. (2019). Longitudinal evolution of the immune suppressive glioma microenvironment in different synchronous lesions during treatment. Neuro-Oncol. Adv..

