# Supplementary material for: Sustained Accumulation of Blood-Derived Macrophages in the Immune Microenvironment of Patients with Recurrent Glioblastoma after Therapy
Source: Cancers (Basel). 2021 Dec 7;13(24):6178. doi: 10.3390/cancers13246178 (PMC8699781; doi:10.3390/cancers13246178)
Supplement: Supplementary file 1 [file cancers-13-06178-s001.zip › cancers-1479310-supplementary.pdf]

cancers

MDPI

Supplementary Materials

Sustained Accumulation of Blood-Derived Macrophages in the Immune Microenvironment of Patients with Recurrent Glioblastoma after Therapy

Sara Magri, Beatrice Musca, Camilla Bonaudo, Ada Tushe, Maria Giovanna Russo, Elena Masetto, Vittorina Zagonel, Giuseppe Lombardi, Alessandro Della Puppa and Susanna Mandruzzato

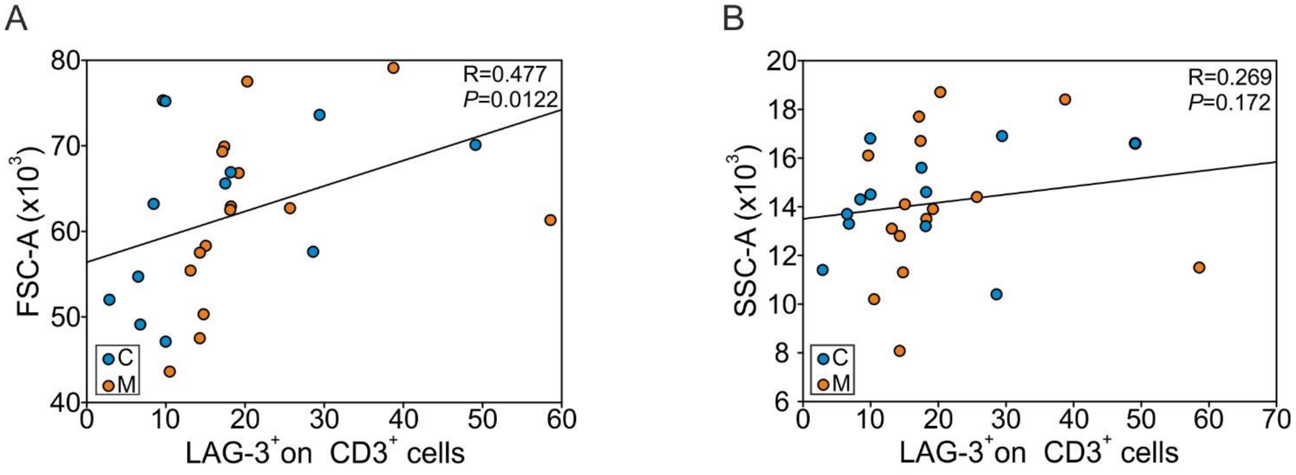

Figure S1. Correlation between LAG-3 expression and morphological parameters in T cells infiltrating GBM TME. (**A–B**) Correlation between LAG-3 expression on CD3$^+$ cells and FSC-A (**A**) and SSC-A (**B**) in primary GBM patients. Spearman's rank-order correlation on 27 paired samples (central layer: n = 12; marginal layer: n = 15).

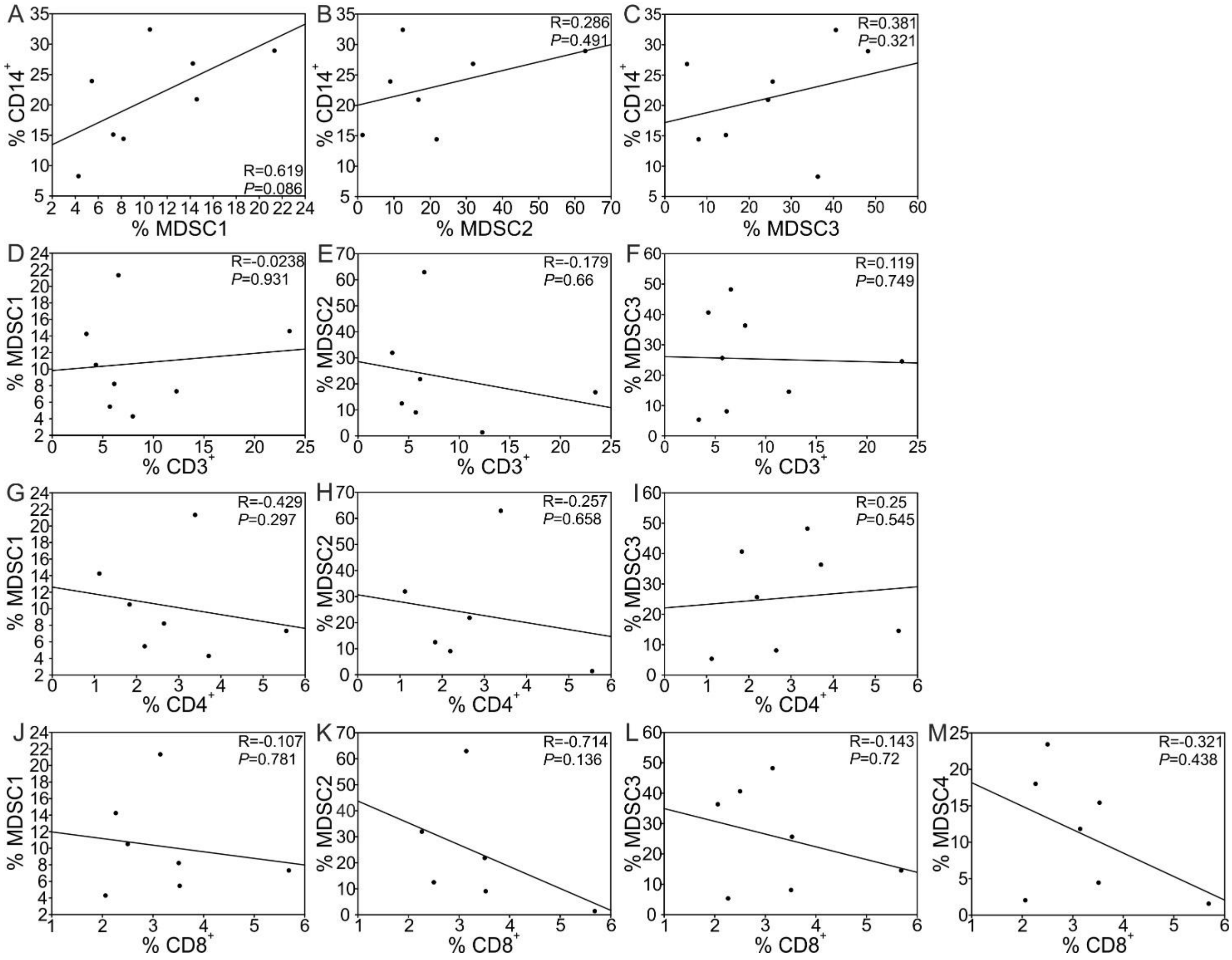

Figure S2. Correlation between circulating myeloid subsets and tumor-infiltrating lymphocytes in relapsing GBM. (**A–C**) Correlation between the percentages of monocytes and MDSC1 (**A**), MDSC2 (**B**) and MDSC3 (**C**) among PBMCs in recurrent patients. Spearman's rank-order correlation on 8 (**A–C**) and 7 (**B**) paired samples. (**D–F**) Correlation between the percentage of tumor-infiltrating $CD3^+$ cells and either circulating MDSC1 (**D**), MDSC2 (**E**) and MDSC3 (**F**). Spearman's rank-order correlation on 8 (**D–F**) and 7 (**E**) paired samples. (**G–I**) Correlation between the percentage of tumor-infiltrating $CD4^+$ cells and either circulating MDSC1 (**D**), MDSC2 (**E**) and MDSC3 (**F**). Spearman's rank-order correlation on 7 (**G–I**) and 5 (**H**) paired samples. (**J–M**) Correlation between the percentage of tumor-infiltrating $CD8^+$ cells and either circulating MDSC1 (**J**), MDSC2 (**K**), MDSC3 (**L**) and MDSC4 (**M**). Spearman's rank-order correlation on 7 (**J–M**) and 6 (**K**) paired samples.
